# The Combination of Neutrophil–Lymphocyte Ratio and Platelet–Lymphocyte Ratio with Liquid Biopsy Biomarkers Improves Prognosis Prediction in Metastatic Pancreatic Cancer

**DOI:** 10.3390/cancers13061210

**Published:** 2021-03-10

**Authors:** Marta Toledano-Fonseca, M. Teresa Cano, Elizabeth Inga, Auxiliadora Gómez-España, Silvia Guil-Luna, María Victoria García-Ortiz, Rafael Mena-Osuna, Juan R. De la Haba-Rodriguez, Antonio Rodríguez-Ariza, Enrique Aranda

**Affiliations:** 1 Maimonides Biomedical Research Institute of Cordoba (IMIBIC), 14004 Córdoba, Spain; marta.toledano@imibic.org (M.T.-F.); v22gulus@uco.es (S.G.-L.); mvictoria.garcia@imibic.org (M.V.G.-O.); b72meosr@uco.es (R.M.-O.); juanr.delahaba.sspa@juntadeandalucia.es (J.R.D.l.H.-R.); earandaa@seom.org (E.A.); 2 Cancer Network Biomedical Research Centre (CIBERONC), 28029 Madrid, Spain; 3 Andalusia-Roche Network Mixed Alliance in Precision Medical Oncology, 41092 Sevilla, Spain; mteresa.cano.sspa@juntadeandalucia.es (M.T.C.); elizabeth.inga.sspa@juntadeandalucia.es (E.I.); auxiliadora.gomez.sspa@juntadeandalucia.es (A.G.-E.); 4 Medical Oncology Department, Reina Sofía University Hospital, 14004 Córdoba, Spain; 5 Department of Medicine, Faculty of Medicine, University of Córdoba, 14004 Córdoba, Spain

**Keywords:** NLR, PLR, circulating tumor DNA, pancreatic ductal adenocarcinoma, RAS mutation, neutrophil elastase

## Abstract

**Simple Summary:**

Liquid biopsy is a noninvasive approach that provides tumor molecular profiling. On the other hand, the vast majority of pancreatic tumors are pancreatic ductal adenocarcinomas (PDAC), which are characterized by pronounced inflammation. Therefore, we hypothesized that the combination of biomarkers of systemic inflammation, such as the neutrophil-to-lymphocyte-ratio (NLR) and platelet-to-lymphocyte ratio (PLR), with liquid biopsy-based biomarkers may increase their clinical usefulness. Our study shows that combining NLR, PLR, and the standard PDAC marker CA19-9 with circulating cell-free DNA and circulating RAS-mutated DNA outperforms traditional clinical tools for the clinical management of metastatic PDAC patients.

**Abstract:**

Pancreatic ductal adenocarcinoma (PDAC) is an aggressive cancer with a highly inflammatory microenvironment and liquid biopsy has emerged as a promising tool for the noninvasive analysis of this tumor. In this study, plasma was obtained from 58 metastatic PDAC patients, and neutrophil–lymphocyte ratio (NLR), platelet–lymphocyte ratio (PLR), circulating cell-free DNA (cfDNA) concentration, and circulating RAS mutation were determined. We found that NLR was significantly associated with both overall survival (OS) and progression-free survival. Remarkably, NLR was an independent risk factor for poor OS. Moreover, NLR and PLR positively correlated, and combination of both inflammatory markers significantly improved the prognostic stratification of metastatic PDAC patients. NLR also showed a positive correlation with cfDNA levels and RAS mutant allelic fraction (MAF). Besides, we found that neutrophil activation contributed to cfDNA content in the plasma of metastatic PDAC patients. Finally, a multi-parameter prognosis model was designed by combining NLR, PLR, cfDNA levels, RAS mutation, RAS MAF, and CA19-9, which performs as a promising tool to predict the prognosis of metastatic PDAC patients. In conclusion, our study supports the idea that the use of systemic inflammatory markers along with circulating tumor-specific markers may constitute a valuable tool for the clinical management of metastatic PDAC patients.

## 1. Introduction

Pancreatic cancer is among the major lethal cancers, with five-year survival of around 5%, and it is the fourth highest cause of cancer mortality in Europe, with more than 95,000 deaths annually [1,2]. Improved survival rates have been achieved in the most common cancers, but pancreatic cancer death rate is increasing [3]. This dismal prognosis is mainly due to advanced stage diagnosis and resistance to therapy. More than 90% of pancreatic tumors are pancreatic ductal adenocarcinomas (PDAC) and the vast majority of deaths are associated with this rapidly progressive and highly aggressive tumor type.

Inflammation plays a critical role in the development and progression of many types of cancer. PDAC is characterized by pronounced inflammation and desmoplasia, leading to hypoxia, metabolic reprogramming, and immune suppression that ultimately promotes tumor growth and metastasis [4]. The neutrophil-to-lymphocyte ratio (NLR) and platelet-to-lymphocyte ratio (PLR) have been proposed as markers of systemic inflammatory response in several solid tumors [5]. Platelets are mainly protumorigenic and neutrophils acquire protumorigenic properties upon recruitment to the tumor microenvironment [6]. Accordingly, levels of circulating neutrophils are elevated in PDAC patients and increased levels of neutrophils infiltrating pancreatic tumors have been reported to correlate with a poor clinical outcome [7,8]. These studies support the theory that neutrophils play a role in inflammation-driven pancreatic tumorigenesis. Besides, low NLR and PLR have been associated with longer overall survival (OS) and progression-free survival (PFS) in pancreatic cancer [9,10].

KRAS oncogene, which is mutationally activated in the vast majority of pancreatic ductal tumors [11], is involved in the release by tumor cells of inflammatory cytokines; the recruitment of immune cells with protumoral activity, thereby promoting an inflamed tumor stroma; and the progression and invasion of PDAC [4,12,13,14]. We have previously reported that RAS mutation status and mutational load in circulating cell-free DNA are independent risk factors for OS in metastatic PDAC patients. Moreover, higher cell-free DNA (cfDNA) concentration and fragmentation levels were also associated with poorer survival [15].

Although NLR and PLR have been shown to be of prognostic value in PDAC, neutrophils, platelets, and lymphocyte counts are affected by other factors, including chemotherapy toxicity [16]. It is necessary to strengthen the clinical usefulness of these biomarkers of systemic inflammation with the information provided by noninvasive tumor biomarkers. Therefore, the present study was aimed at evaluating the prognostic value of combining NLR and PLR with circulating liquid biopsy markers associated to poor survival outcome in metastatic PDAC patients.

## 2. Materials and Methods

### 2.1. Patients

A total of 58 patients with a diagnosis of metastatic PDAC were included in this study. Patients were enrolled from the Reina Sofía Hospital (Córdoba, Spain) from 2017 to 2019 with the following inclusion criteria: older than 18 years with histologically confirmed metastatic PDAC and without previous chemotherapy or radiotherapy. The study was approved by the ethics committee of our hospital and written informed consent was obtained from all patients before enrollment. Patients’ characteristics are summarized in Table 1.

### 2.2. Procedures for Sample Analyses

Platelets, neutrophils, and lymphocyte were measured routinely in the clinical laboratory department of our hospital. NLR and PLR were calculated by dividing absolute neutrophil count and platelets count by the absolute lymphocyte count, respectively. Tumor biomarker CA19-9 was also measured in the clinical laboratory department of our hospital and a cut-off value of 45,500 U/mL, previously determined in our research [15], was used.

The analysis of cell-free DNA (cfDNA) was performed as previously described [15]. In brief, plasma was obtained from 10 mL of peripheral blood before treatment and cfDNA was extracted from 3 mL of plasma with the QIAamp Circulating Nucleic Acid Kit and the vacuum system QIAvac 24 Plus (Qiagen, Hilden, Germany). The Quantus fluorometer (Promega, Madison, WI, USA) and the Agilent 2200 TapeStation system (Agilent, Santa Clara, CA, USA) with the High Sensitivity D1000 ScreenTape assay were used for measuring cfDNA concentration and fragmentation, respectively. The OncoBEAM™ RAS assay (Sysmex Inostics GmbH, Hamburg, Germany) was used for the analysis of RAS mutations in cfDNA and the determination of RAS mutant allelic fraction (MAF) in plasma.

For the quantification of elastase circulating levels, the Human PMN Elastase ELISA Kit (Abcam, Cambridge, UK) was used following the manufacturer’s instructions.

### 2.3. Statistical Analyses

SPSS Statistic 20.0.0, GraphPad Prism 7.0 Software and R Software 4.0.0 were used for data analysis. OS was computed from the time of diagnosis until the date of death and PFS was determined as the time from the start of therapy until documented disease progression. Estimation of survival rates and the identification of prognostic variables were performed with the Kaplan–Meier method and the log-rank test, respectively. The optimal cut-off values were selected with the SurvivalROC package based on the time-dependent ROC curve and were selected by minimalizing the sum of false negative and false positive rates [17]. The cut-off value with prognostic relevance for OS was also tested for prognosis of PFS. Mann–Whitney test was used to compare differences between two groups and ANOVA test was performed when comparing more than two groups. Multivariate analysis with Cox proportional hazards regression was used to determine independent prognostic factors. Correlation analyses were performed using Pearson’s correlation coefficient. Data in graphs are represented as mean ± standard deviation. All results were considered statistically significant when *p* < 0.05.

## 3. Results

### 3.1. Clinicopathological Characteristics of Patients

**Figure 1:**
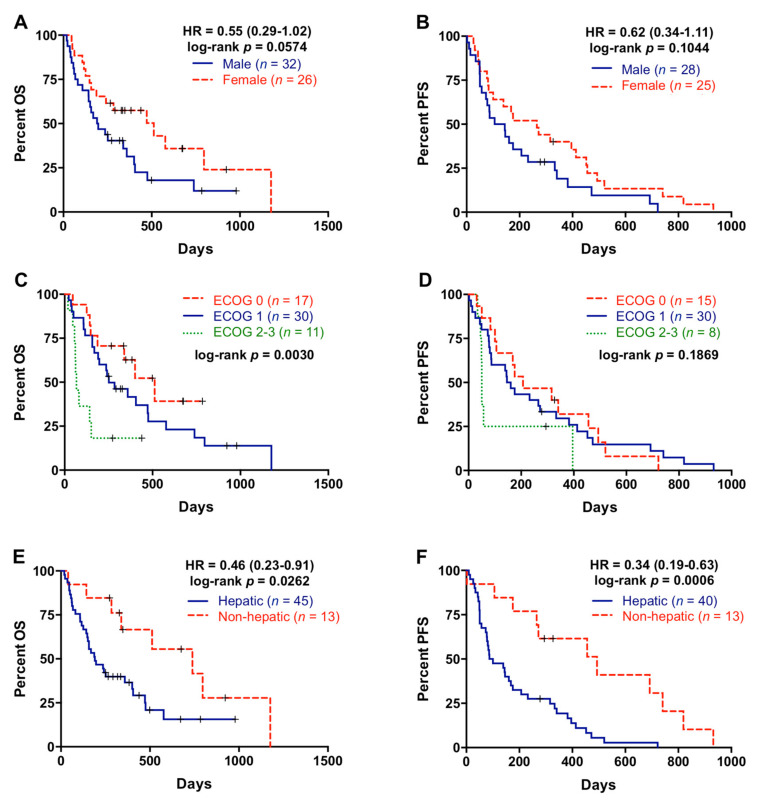
Overall survival and progression-free survival rates according to the clinical characteristics of the patients. (**A**) Overall survival (OS) according to gender; (**B**) progression-free survival (PFS) according to gender; (**C**) OS according to EGOG; (**D**) PFS according to ECOG; (**E**) OS according to metastatic location; (**F**) PFS according to metastatic location.

Fifty-eight patients were included in the study between 2017 and 2019 (baseline characteristics are summarized in Table 1). Most patients (81%) had a good baseline ECOG (ECOG 0–1) and the majority of them (75.8%) received first-line gemcitabine-based regimes. As shown in Figure 1A,B, there was a trend towards females (*n* = 26) having better OS than males (*n* = 32) (193.5 versus 310 days; *p* = 0.0574) and also better PFS, although not statistically significant (125 versus 265 days; *p* = 0.1044). Patients included in the study ranged in age from 40 to 84 years, with a median of 65 years of age. When patients were stratified according to age (60 years), no differences in OS and PFS were found (Table 2). ECOG was related with better OS (*p* = 0.0030), whereas there were no significant association between ECOG and PFS (*p* = 0.1869) (Figure 1C,D).

All PDAC patients had distant metastases at diagnosis, the liver being the most frequent site of metastasis (77.6%, Table 1). Patients with metastatic lesions located in the liver had significantly poorer OS and PFS rates (*p*= 0.0262 and *p* = 0.0006, respectively) compared with patients with metastasis affecting other organs (Figure 1E,F). On the contrary, there was no significant association between number of metastatic lesions and OS or PFS (Table 2). Primary tumor sites were tail, body, and head of pancreas in 27.6%, 41.4%, and 29.3% of patients, respectively (Table 1), but primary tumor location was not significant related with OS or PFS (Table 2).

### 3.2. NLR and PLR Are Prognostic Markers in Metastatic PDAC Patients

**Figure 2:**
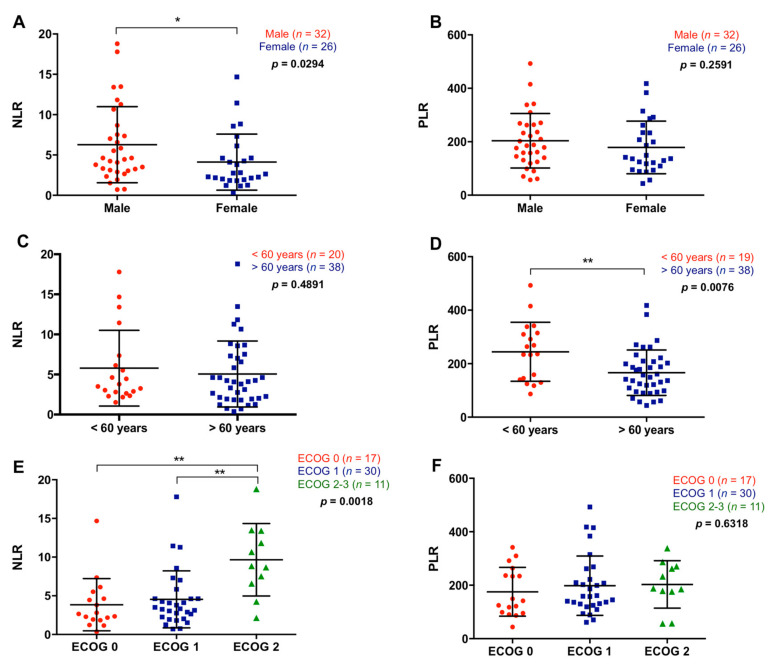
Association between neutrophil–lymphocyte ratio and platelet–lymphocyte ratio with the clinical characteristics of the patients. (**A**) Neutrophil–lymphocyte ratio (NLR) and (**B**) platelet–lymphocyte ratio (PLR) in male or females patients, (**C**) NLR and (**D**) PLR in patients younger or older than 60 years, (**E**) NLR and (**F**) PLR according to the ECOG (* *p* < 0.05, ** *p* < 0.01).

NLR and PLR were analyzed in 58 metastatic PDAC patients. One patient with an ultra-high platelets count was excluded from the PLR analysis. The median NLR in plasma of metastatic PDAC patients was 3.94 (range 0.38–18.8) and the median PLR was 176.07 (range 43.59–492.86). There was a significant association of high NLR with male gender (*p* = 0.0294), while no relation was found between PLR and gender (*p* = 0.2591) (Figure 2A,B). On the other hand, although no relation was found between NLR and age (*p* = 0.4891), patients older than 60 years showed a significantly lower PLR than those younger (153.39 versus 236.47; *p* = 0.0076) (Figure 2C,D). Besides, as shown in Figure 2E,F, higher NLR, but not PLR, was associated with worse ECOG (ECOG 2–3) (NLR: *p* = 0.0018; PLR: *p* = 0.6318).

**Figure 3:**
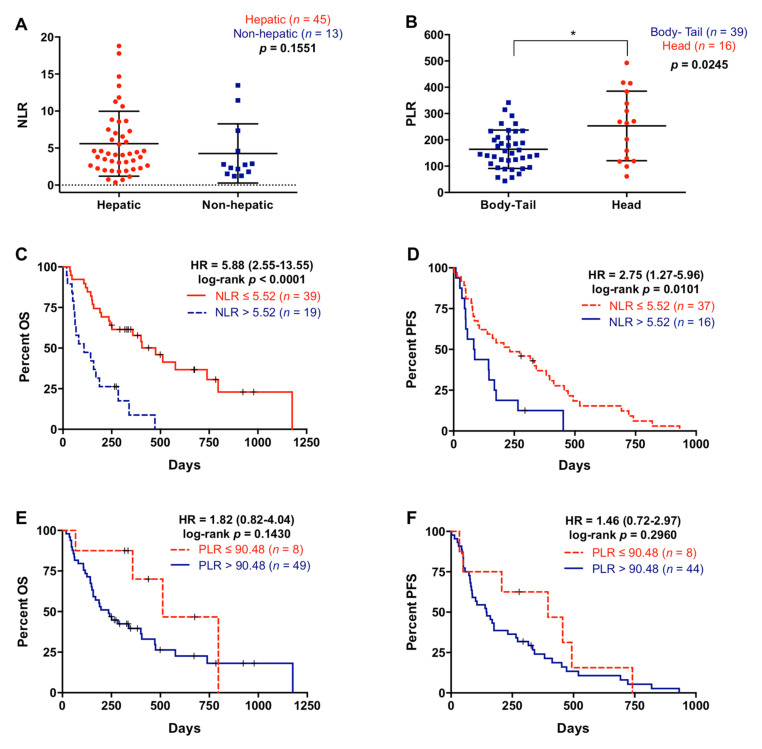
Neutrophil–lymphocyte ratio and platelet–lymphocyte ratio in metastatic pancreatic ductal adenocarcinoma patients. (**A**) Neutrophil–lymphocyte ratio (NLR) in patients with liver metastasis or metastases elsewhere, (**B**) platelet–lymphocyte ratio (PLR) in patients with primary tumor located in the body-tail or the head of the pancreas, (**C**) overall survival (OS) according to NLR (cut-off: 5.52), (**D**) progression-free survival (PFS) according to NLR (cut-off: 5.52), (**E**) OS according to PLR (cut-off: 90.48), (**F**) PFS according to PLR (cut-off: 90.48) (* *p* < 0.05).

NLR was not significantly associated with primary tumor site (*p* = 0.7859) or number of metastasis (*p* = 0.2859), although NLR showed a trend towards higher values in patients with metastatic lesions located in the liver compared with patients with metastasis affecting other organs (*p* = 0.1551) (Figure 3A). Contrarily, PLR was not associated with metastatic location (*p* = 0.7558) or the number of metastasis (*p* = 0.7653), but patients with the primary tumor in the head of the pancreas showed higher PLR compared with tumors in the body/tail location (266.2 versus 149.16; *p* = 0.0245) (Figure 3B). Patients with higher NLR (>5.52) had significantly poorer OS (108 versus 335 days; *p* < 0.0001) and PFS (85 versus 232 days; *p* = 0.0101) rates (Figure 3C,D). Moreover, multivariate analysis revealed that NLR was an independent prognostic factor for OS (HR 2.466, 95% CI 1.246–4.880; *p* = 0.010), along with ECOG and RAS mutation status (Table 3). Also, although not significant, patients with higher PLR (>90.48) showed a trend towards poorer OS (236 versus 399 days; *p* = 0.1430) and PFS (145 versus 337 days; *p* = 0.2960) (Figure 3E,F).

### 3.3. Combined Analysis of NLR and PLR Improves the Prognostic Accuracy in Patients with Metastatic PDAC 

**Figure 4:**
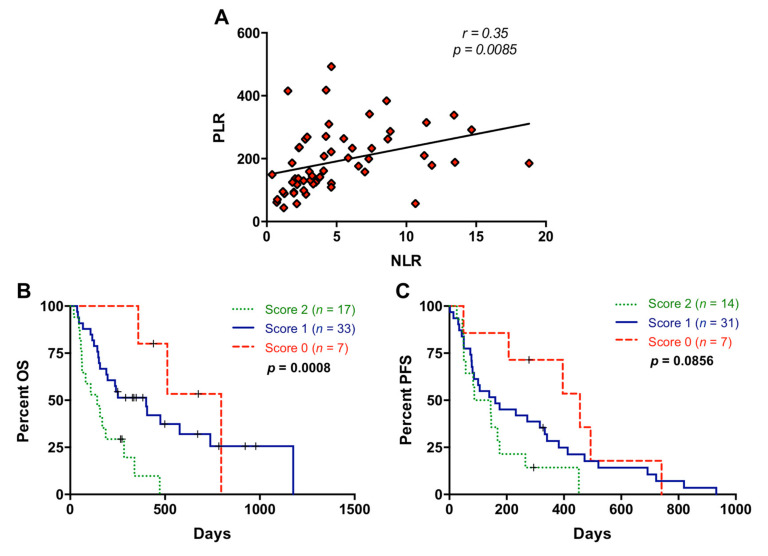
Association between neutrophil–lymphocyte ratio and platelet–lymphocyte ratio. (**A**) Correlation between neutrophil–lymphocyte ratio (NLR) and platelet–lymphocyte ratio (PLR) values; (**B**) overall survival (OS) according to the NLR and PLR combination (score 2 compared to score 0: *p* = 0.0004, score 2 compared to score 1: *p* = 0.0040); (**C**) progression-free survival (PFS) according to the NLR and PLR combination (score 2 compared to score 0: *p* = 0.0097; score 2 compared to score 1: *p* = 0.1463) (score 2, positive for both markers; score 1, positive for one of them; score 0: negative for both markers).

A significant positive correlation between NLR and PLR was observed (r = 0.35; *p* = 0.0085) (Figure 4A). Notably, the combination of NLR and PLR improved the prognostic classification of metastatic PDAC patients. For this combined analysis, positive or negative values were assigned when NLR or PLR values were above (positive) or below (negative) the cut-off for prognostic value in OS, and scores 0, 1, and 2 were defined as negative for both markers, positive for one of them, and positive for both markers, respectively. As shown in Figure 4B, those patients with score 2 showed a highly significant poorer survival than those patients with score 0 or 1 in the Kaplan–Meier analysis (*p* = 0.0004 and *p* = 0.0040, respectively). In contrast, the combination of NLR and PLR did not significantly improve prognosis accuracy for PFS (*p* = 0.0856) (Figure 4C).

### 3.4. The Combination of NLR and cfDNA Values Significantly Improves Prognostic Stratification of Metastatic PDAC Patients

**Figure 5:**
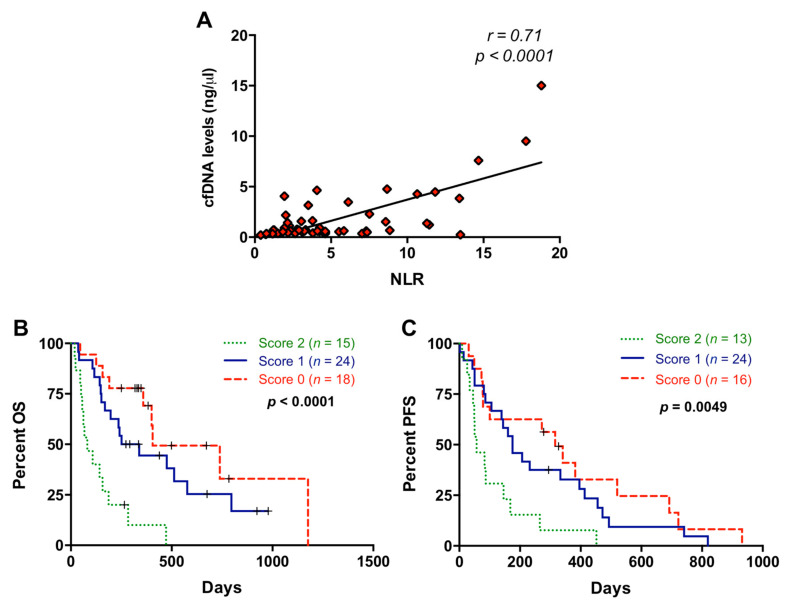
Association between neutrophil–lymphocyte ratio and cell-free DNA concentration. (**A**) Correlation between neutrophil–lymphocyte ratio (NLR) values and circulating cell-free DNA (cfDNA) levels; (**B**) overall survival (OS) according to the NLR and cfDNA combination (score 2 compared to score 0: *p* = 0.0001, score 2 compared to score 1: *p* = 0.0008); (**C**) progression-free survival (PFS) according to the NLR and cfDNA combination (score 2 compared to score 0: *p* = 0.0037; score 2 compared to score 1: *p* = 0.0119) (score 2, positive for both markers; score 1, positive for one of them; score 0: negative for both markers).

cfDNA concentration was measured in our patient cohort (median: 32.6 ng/mL; range: 10–700 ng/mL). A high positive correlation (r = 0.71; *p* < 0.0001) was found between NLR and cfDNA concentration (Figure 5A). No correlation was found between PLR and cfDNA concentration (*p* = 0.8205). However, a negative correlation (r = −0.30; *p* = 0.0244) was found between PLR and cfDNA size (Figure S1). No significant association was found between NLR and cfDNA fragmentation (*p* = 0.4381).

We previously reported that higher levels of cfDNA were significantly associated with shorter OS and PFS in metastatic PDAC patients and a cut-off for cfDNA concentration (26.46 ng/mL) was determined [15]. Using this cut-off, patients included in the present study also showed differences in OS (172 versus 339 days; *p* = 0.0169) and a trend for PFS (122.5 versus 278 days; *p* = 0.0664) according to cfDNA concentration. When NLR and cfDNA levels were combined according to the scoring system described above, patients with score 2 showed significantly shorter OS than patients with score 0 or score 1 (*p* = 0.0001 and *p* = 0.0008, respectively) (Figure 5B). We also found an improvement in the prognostic stratification of patients according to PFS when NLR and cfDNA levels were combined (score 2 versus score 0: *p* = 0.0037 and score 2 versus score 1: *p*= 0.0119) (Figure 5C).

**Figure 6:**
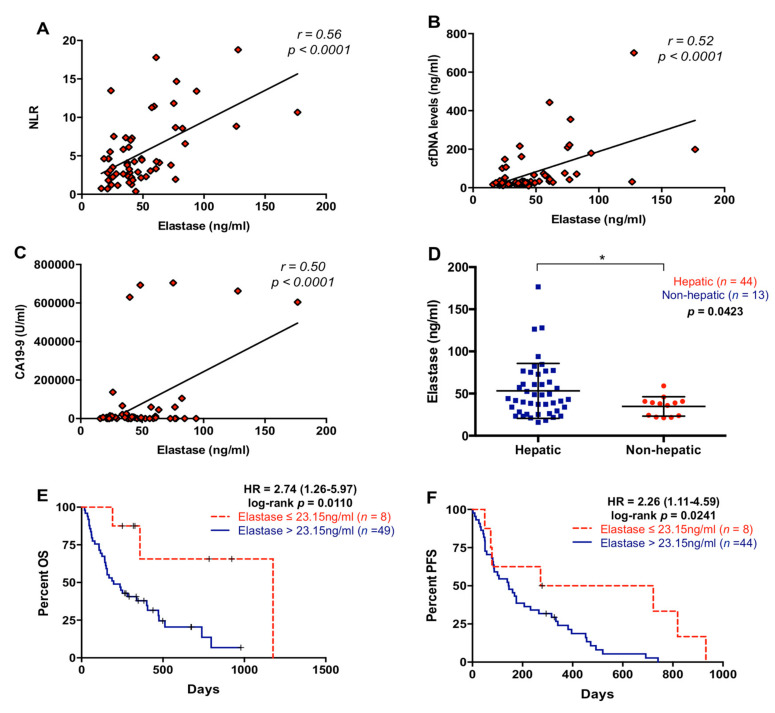
Circulating levels of neutrophil elastase in metastatic pancreatic ductal adenocarcinoma patients. (**A**) Correlation between plasma levels of neutrophil elastase and neutrophil–lymphocyte ratio (NLR); (**B**) correlation between plasma levels of neutrophil elastase and cell-free DNA (cfDNA); (**C**) correlation between plasma levels of neutrophil elastase and CA19-9; (**D**) plasma levels of neutrophil elastase in pancreatic ductal adenocarcinoma patients with metastatic lesions in the liver or elsewhere; (**E**) overall survival (OS) according to circulating levels of neutrophil elastase (cut-off: 23.15 ng/mL); (**F**) progression-free survival (PFS) according to circulating levels of neutrophil elastase (cut-off: 23.15 ng/mL) (* *p* < 0.05).

Since NLR and cfDNA concentration were highly correlated, we next measured neutrophil elastase in plasma as a marker of NETosis, which is a process involving the formation of neutrophil extracellular traps (NETs), to determine whether neutrophil activation contributes to cfDNA content in plasma of metastatic PDAC patients. As shown in Figure 6A–C, elastase concentration in plasma positively correlated with NLR (r = 0.5618; *p* < 0.0001), cfDNA concentration (r = 0.5246; *p* < 0.0001), and CA19-9 (r = 0.4995; *p* < 0.0001). Elastase concentration was higher in patients with liver metastasis (*p* = 0.0423, Figure 6D), in agreement with higher (although not statistically significant) NLR values in patients with hepatic metastases (Figure 3A), and also with our previously reported trend towards higher cfDNA levels in patients with hepatic lesions [15]. However, there was no relation between elastase levels and primary tumor location (*p* = 0.9890) or number of metastasis (*p* = 0.7515). Moreover, high elastase concentration in plasma (>23.15 ng/mL) was a prognostic factor of worse OS (*p* = 0.0110) and PFS (*p* = 0.0241) (Figure 6E,F).

### 3.5. NLR Is Related to RAS Mutational Status in cfDNA of Metastatic PDAC Patients

**Figure 7:**
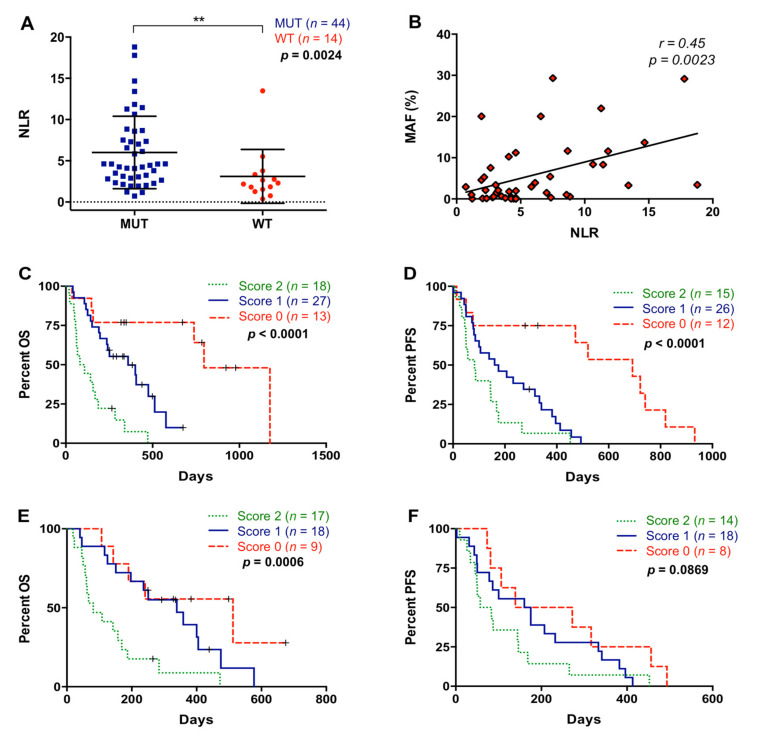
Association between neutrophil–lymphocyte ratio and plasma RAS mutation. (**A**) Neutrophil–lymphocyte ratio (NLR) according to RAS mutational status in plasma; (**B**) correlation between NLR values and RAS mutant allelic fraction (MAF) in plasma; (**C**) overall survival (OS) according to the NLR and RAS mutational status combination (score 2 compared to score 0: *p* < 0.0001; score 2 compared to score 1: *p* = 0.0003); (**D**) progression-free survival (PFS) according to the NLR and RAS mutational status combination (score 2 compared to score 0: *p* = 0.0003; score 2 compared to score 1: *p* = 0.0533); (**E**) OS according to the NLR and MAF combination (score 2 compared to score 0: *p* = 0.0029; score 2 compared to score 1: *p* = 0.0037); (**F**) PFS according to the NLR and MAF combination (score 2 compared to score 0: *p* = 0.0420; score 2 compared to score 1: *p* = 0.3008) (score 2, positive for both markers; score 1, positive for one of them; score 0, negative for both markers) (** *p* < 0.01).

Analysis of RAS mutation status in cfDNA was performed in 58 metastatic PDAC patients included in this study. RAS mutation was detected in 75.9% (44/58) of plasma samples (Table 1). As shown in Figure 7A, NLR was significantly higher in those patients in which plasma RAS mutations were detected (4.53 versus 2.24; *p* = 0.0024). Moreover, a positive correlation between NLR and the RAS MAF was found (r = 0.4481; *p* = 0.0023) (Figure 7B). We have previously reported [15] that the detections of RAS mutations and RAS mutational load in cfDNA were related to shorter OS and PFS in metastatic PDAC patients and a cut-off for MAF was determined (RAS MAF cut-off: 0.351%). Patients included in the present study also showed poor OS (193.5 versus 510 days; *p* = 0.0003) and PFS (122.5 versus 472 days; *p* < 0.0001) when RAS mutation was detected in plasma. Moreover, patients with higher RAS MAF in cfDNA had worse OS (163 versus 333.5 days; *p* = 0.0365) and a trend towards poorer PFS (87 versus 175 days; *p* = 0.0818). As shown in Figure 7C, when NLR and RAS mutation status in plasma were combined, patients with score 2 showed poorer OS compared with patients with score 0 and score 1 (*p* < 0.0001 and *p* = 0.0003, respectively). Similarly, this combination of markers also better stratified patients for PFS (score 2 versus score 0: *p* = 0.0003 and score 2 versus score 1: *p* = 0.0533) (Figure 7D). When NLR was combined with RAS MAF (Figure 7E), an improved stratification of RAS-mutated patients for OS (score 2 versus 0, *p* = 0.0029; score 2 versus 1, *p* = 0.0037) but not for PFS (*p* = 0.0869) was observed (Figure 7F). No significant association was found between PLR and RAS mutation (*p* = 0.5071) or RAS mutational load (*p* = 0.6854) in plasma.

### 3.6. Multiple Blood-Based Biomarkers Improve the Prognostic Stratification of Metastatic PDAC Patients

**Figure 8:**
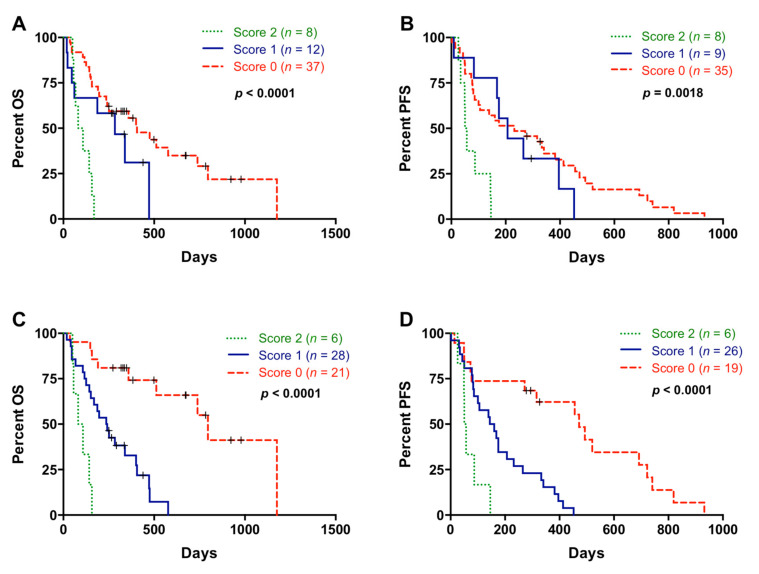
Multiple blood-based biomarkers for the prognosis of metastatic pancreatic ductal adenocarcinoma patients. (**A**) Overall survival (OS) according to neutrophil–lymphocyte ratio (NLR) and CA19-9 combination (score 2 compared to score 0: *p* < 0.0001; score 2 compared to score 1: *p* = 0.0226); (**B**) progression-free survival (PFS) according to NLR and CA19-9 combination (score 2 compared to score 0: *p* = 0.0016; score 2 compared to score 1: *p* = 0.0021) (score 2, positive for both markers; score 1, positive for one of them; score 0, negative for both markers); (**C**) OS according to the combination of multiple blood-based biomarkers (NLR, platelet–lymphocyte ratio (PLR), cell-free DNA (cfDNA) concentration, RAS status, RAS mutant allelic fraction (MAF) and CA19-9) (score 2 compared to score 0: *p* < 0.0001; score 2 compared to score 1: *p* = 0.0026); (**D**) PFS according to the combination of multiple blood-based biomarkers (NLR, PLR, cfDNA concentration, RAS status, RAS MAF, and CA19-9) (score 2 compared to score 0: *p* = 0.0008; score 2 compared to score 1: *p* = 0.0086) (score 2, positive for all markers; score 1, positive for 3, 4 or 5 markers; score 0, positive for 1 or 2 markers or negative for all of them).

Patients included in this analysis showed differences in OS (125 versus 284 days; *p* = 0.0223) and in PFS (72 versus 203.5 days; *p* = 0.0110) according to CA19-9 levels. CA19-9 levels and NLR showed a positive correlation (r = 0.3684; *p* = 0.0048; Figure S2) and the combination of both showed an improvement in patient stratification for OS (score 2 versus score 0: *p* < 0.0001 and score 2 versus score 1: *p* = 0.0226) and PFS (score 2 versus score 0: *p* = 0.0016 and score 2 versus score 1: *p* = 0.0021) (Figure 8A,B).

Next, a combination of multiple blood-based biomarkers (NLR, PLR, cfDNA concentration, RAS status, RAS MAF, and CA19-9) was used to improve the prognostic stratification of metastatic PDAC patients. In this case, score 2 was defined as positive for all markers; score 1 positive for 3, 4, or 5 markers; and score 0 positive for 1 or 2 markers or negative for all of them. As shown in Figure 8C, patients with score 2 had a very short OS outcome compared with patients with score 1 (*p* = 0.0026), and especially compared with patients with score 0 (*p* < 0.0001). In regard to PFS, this combination of multiple blood-based biomarkers also efficiently stratified patients into dismal (score 2), poor (score 1), and good (score 0) prognosis (Figure 8D).

## 4. Discussion

Infiltration of immune cells in PDAC tumors is highly abundant, contributing to immune evasion and chemotherapy resistance [4]. In the present study, we described the utility of NLR and PLR along with others circulating tumor-specific markers to evaluate the prognosis in metastatic PDAC patients.

Previous reports have related high NLR and PLR values with poor prognosis in advanced pancreatic cancer [16,18,19,20]. However, most of the studies that have related high PLR values with poor OS involved locally advanced patients [20,21,22], who were not included in the present study. In this regard our analysis showed that metastatic PDAC patients with higher NLR values had significantly poorer OS and PFS rates, whereas both NLR and PLR were associated with poor-prognosis clinical features. Thus, higher NLR values were related with male gender and higher ECOG status. This is in agreement with other cancer studies, in which higher NLR values were reported in male cancer patients [23,24] and patients with high ECOG status [21]. In addition, those patients younger than 60 years had higher PLR values than older patients, likely because aging is known to be accompanied by a decrease in platelet count [25]. Further, patients with a primary tumor located in the head of the pancreas showed higher PLR values than those patients with a tumor in the body/tail of this organ. 

Cancer cells can activate platelets leading to pro-cancerous effects. Activated platelets participate in the regulation of inflammation, releasing proinflammatory cytokines, and in modulating tumor microenvironment by recruiting leukocytes, including neutrophils. Moreover, activated platelets participate in tumor immune evasion by releasing transforming growth factor β (TGF-β), which is a cytokine with a potent immunosuppressive activity. Besides, TGF-β participates in the transition of tumor-associated neutrophils from an antitumorigenic (N1) towards a protumorigenic (N2) phenotype [4]. Furthermore, activated platelets have been implicated in the formation of NETs, with a positive feedback loop, because NETs in turn promote platelet activation [26,27]. Therefore, the platelet–neutrophil crosstalk plays an important role in the development and progression of cancer. In this regard, our analysis indicated that NLR and PLR positively correlatde and the combination of both factors increased their prognostic value. On the other hand, platelets and neutrophils have been related with the metastatic process [28,29,30], and our results confirmed that NLR was associated with liver metastasis in PDAC patients, in agreement with a previous report [31].

There is increasing evidence connecting KRAS mutations with tumor-promoting inflammation in several human cancers, including PDAC [13,32]. KRAS activation in cancer cells induces the expression and secretion of proinflammatory cytokines, stimulating the recruitment of neutrophils to the tumor [6]. On the other hand, gene dosage of mutant KRAS has an important role in PDAC biology [33], and we and others have recently reported the correlation of KRAS MAF in cfDNA with clinical stage and outcome in PDAC [8,15,34]. Importantly, in the present study, those patients with RAS-mutated cfDNA had higher NLR values and a positive correlation between NLR and RAS mutational load in cfDNA was found. Furthermore, the combination of NLR with RAS mutational status or load (MAF) in cfDNA greatly improved the prognostic classification of metastatic PDAC patients.

The prognostic significance of cfDNA levels and fragmentation has been previously described in metastatic cancer [35,36,37], including metastatic PDAC [15,38]. Specifically, we have recently reported that higher cfDNA concentration and smaller cfDNA fragment size are associated with poor outcomes in metastatic PDAC patients [15]. In the present study, PLR was negatively correlated with cfDNA fragment size, and this may explain why higher PLR values are associated with more aggressive tumors. Moreover, although apoptosis and necrosis seem to provide most of cfDNA, some stimuli can activate neutrophils leading to DNA release and NETosis [39,40]. A previous study demonstrated a relation between NLR and altered values of cfDNA in endometrial cancer [41]. In this regard, our results showed a high positive correlation between NLR and cfDNA concentration but not cfDNA fragmentation. Moreover, a positive correlation between neutrophil elastase circulating levels and cfDNA concentration was found, suggesting that neutrophil activation significantly contributes to cfDNA content in plasma of metastatic PDAC patients. Besides, the positive correlation found between elastase and CA19-9 suggests that neutrophil activation and NETosis are related with disease progression in metastatic PDAC patients. In fact, higher elastase circulating levels were related with liver metastasis and poor OS and PFS. These findings are consistent with the reported interaction of neutrophils with circulating tumor cells facilitating their contact with hepatic endothelial cells, thus helping cancer cells dissemination and liver metastasis [42,43]. Also, inhibition of NETs has been shown to reduce liver metastasis in a preclinical model of metastatic colorectal cancer [44], while recent studies have suggested that NETs can also contribute to hepatic metastasis in PDAC [45].

Finally, we also showed that the combination of NLR and PLR with cfDNA-based liquid biopsy markers greatly improves prognostic power and provides accurate survival risk stratification. 

## 5. Conclusions

In conclusion, our study supports that the use of NLR and PLR, along with other noninvasive biomarkers in a multi-parameter prognostic model, may constitute a valuable tool for the clinical management of metastatic PDAC patients. Future larger studies are warranted to validate the prognostic value in PDAC patients of this combination of systemic inflammatory and liquid biopsy biomarkers.

## Figures and Tables

**Table 1:** Baseline characteristics of patients.

Patient Characteristics		*n* (%)
Age	<60 years	20 (34.5)
	>60 years	38 (65.5)
Sex	Male	32 (55.2)
	Female	26 (44.8)
ECOG	0	17 (29.3)
	1	30 (51.7)
	2	8 (13.8)
	3	3 (5.2)
1st line treatment	Gemcitabine	2 (3.4)
	Gemcitabine/nab-paclitaxel	38 (65.5)
	Gemcitabine/nab-paclitaxel/FOLFOX	4 (6.9)
	FOLFIRINOX	11 (19)
	No treatment	3 (5.2)
Survival	Alive	17 (29.3)
	Dead	41 (70.7)
Disease progression	Yes	50 (86.2)
	No	3 (5.2)
	Not valuable (No treatment or surgery)	5 (8.6)
Primary tumor location	Tail	16 (27.6)
	Body	24 (41.4)
	Head	16 (27.6)
	No data	2 (3.4)
Number of metastatic lesions	One location	25 (43.1)
	More than one location	33 (56.9)
Metastatic lesions location	Hepatic lesions	45 (77.6)
	Non-hepatic lesions	13 (22.4)
Liquid Biopsy RAS status	RAS mutated	44 (75.9)
	RAS wild-type	14 (24.1)

**Table 2:** Overall survival analysis.

Variables	OS		PFS	
	HR (95%CI)	*p*	HR (95%CI)	*p*
Age				
≤60 years	1.113 (0.584–2.119)	0.7452	0.925 (0.505–1.694)	0.8015
>60 years				
Gender				
Male	0.545 (0.291–1.019)	0.0574	0.615 (0.343–1.106)	0.1044
Female				
ECOG				
0		0.0030		0.1869
1	1.653 (0.789–3.465)		1.083 (0.568–2.065)	
2–3	5.967 (1.86–19.16)		3.166 (0.978–10.25)	
Primary Tumor Location				
Body/Tail	1.48 (0.72–3.04)	0.2884	1.52 (0.74–3.10)	0.2500
Head				
Number of Metastasis				
1	1.5 (0.803–2.801)	0.2035	1.449 (0.811–2.588)	0.2100
≥2				
Metastatic Location				
Hepatic	0.462 (0.234–0.913)	0.0262	0.344 (0.187–0.634)	0.0006
Nonhepatic				
RAS mutation status plasma				
MUT	0.283 (0.141–0.565)	0.0003	0.240 (0.126–0.458)	<0.0001
WT				
NLR				
≤5.52	5.881 (2.552–13.55)	<0.0001	2.754 (1.272–5.962)	0.0101
>5.52				
PLR				
≤90.48	1.816 (0.817–4.035)	0.1430	1.460 (0.718–2.972)	0.2960
>90.48				
cfDNA concentration				
≤26.46 ng/mL	2.173 (1.149–4.107)	0.0169	1.708 (0.964–3.025)	0.0664
>26.46 ng/mL				
MAF				
≤0.351%	2.151 (1.049–4.409)	0.0365	1.859 (0.925–3.737)	0.0818
>0.351%				
CA19-9				
≤45,500 U/mL	3.514 (1.196–10.32)	0.0233	3.508 (1.334–9.227)	0.0110
>45,500 U/mL				

**Table 3:** Multivariate analysis.

Variables	OS		PFS	
	HR (95%CI)	*p*	HR (95%CI)	*p*
ECOG	2.024 (1.207–3.393)	0.008	-	-
Metastatic Location	-	ns	3.150 (1.359–7.305)	0.007
RAS mutation status plasma	6.944 (2.033–23.73)	0.002	7.908 (2.482–25.20)	0.0001
NLR	2.466 (1.246–4.880)	0.010	-	ns

## Data Availability

The data presented in this study are available on reasonable request from the corresponding author.

## Supplementary Materials

The following are available online at https://www.mdpi.com/2072-6694/13/6/1210/s1, Figure S1: Correlation between platelet-lymphocyte ratio (PLR) and circulating cell-free DNA (cfDNA) size, Figure S2: Correlation between neutrophil-lymphocyte ratio (NLR) and CA19-9.
