# Supplementary material for: The Combination of Neutrophil–Lymphocyte Ratio and Platelet–Lymphocyte Ratio with Liquid Biopsy Biomarkers Improves Prognosis Prediction in Metastatic Pancreatic Cancer
Source: Cancers (Basel). 2021 Mar 10;13(6):1210. doi: 10.3390/cancers13061210 (PMC7998484; doi:10.3390/cancers13061210)
Supplement: Supplementary file 1 [file cancers-13-01210-s001.pdf]

The Combination of Neutrophil–Lymphocyte Ratio and Platelet–Lymphocyte Ratio with Liquid Biopsy Biomarkers Improves Prognosis Prediction in Metastatic Pancreatic Cancer

Supplementary Figures:

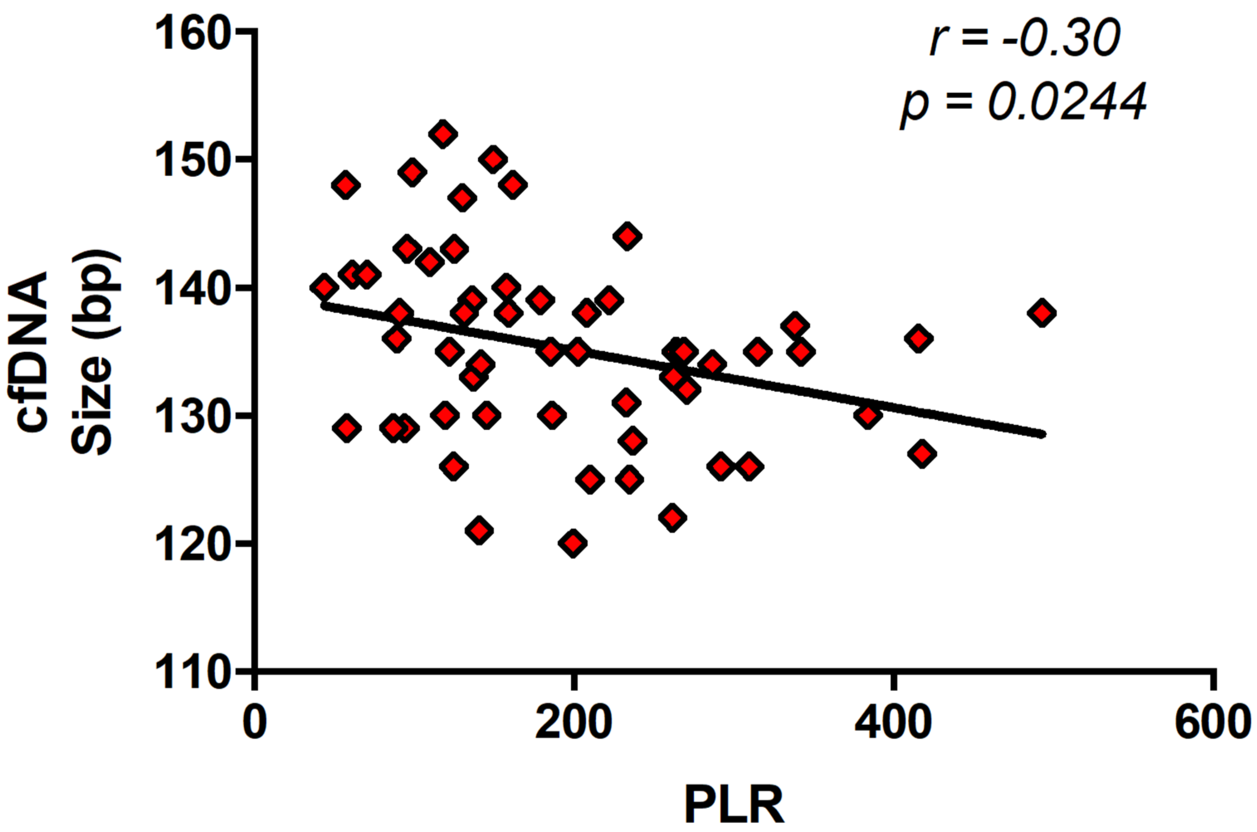

Figure S1. Correlation between platelet-lymphocyte ratio (PLR) and circulating cell-free DNA (cfDNA) size.

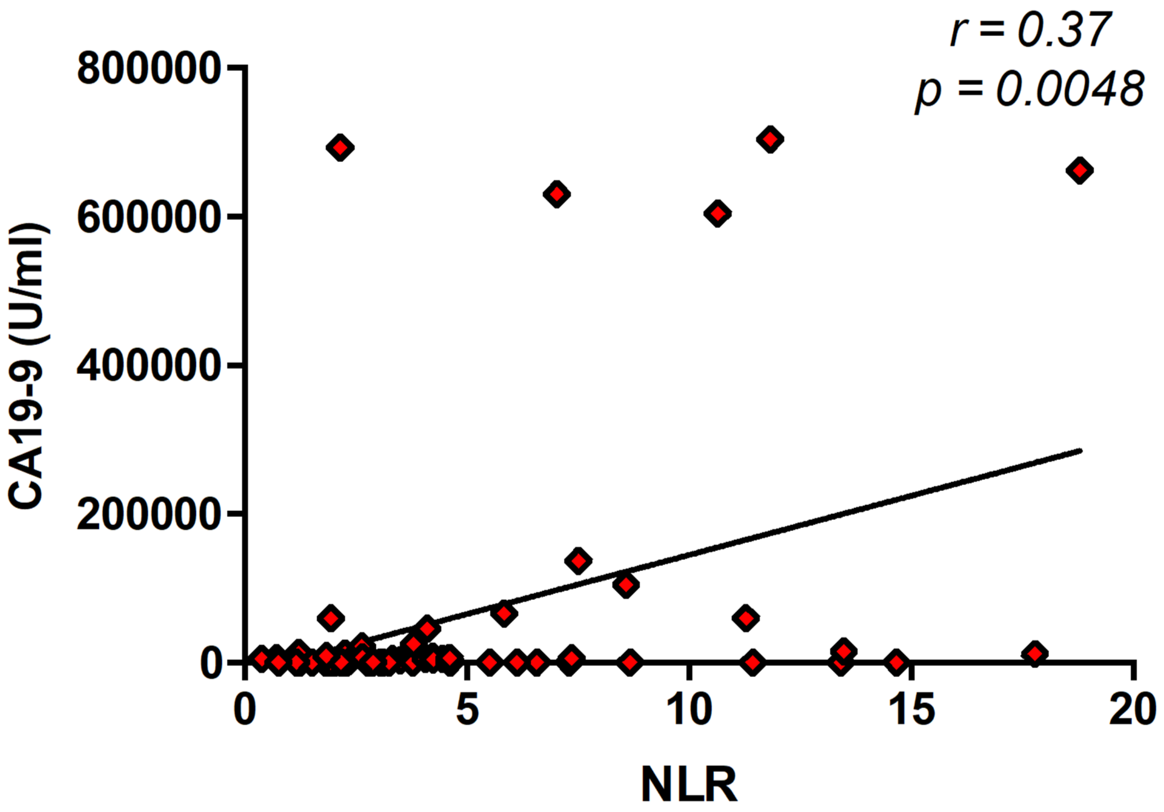

Figure S2. Correlation between neutrophil-lymphocyte ratio (NLR) and CA19-9.
